# Cellulose Structural Changes during Mild Torrefaction of *Eucalyptus* Wood

**DOI:** 10.3390/polym12122831

**Published:** 2020-11-28

**Authors:** Ana Lourenço, Solange Araújo, Jorge Gominho, Dmitry Evtuguin

**Affiliations:** 1 Forest Research Center, School of Agriculture, University of Lisbon, Tapada da Ajuda, 1349-017 Lisboa, Portugal; araujo@isa.ulisboa.pt (S.A.); jgominho@isa.ulisboa.pt (J.G.); 2 CICECO, Chemistry Department, University of Aveiro, Campus de Santiago, P-3810-193 Aveiro, Portugal

**Keywords:** cellulose crystallinity, thermal treatment, *Eucalyptus* wood, WAXS, solid-state ^13^C NMR, FTIR, Py-GC/MS

## Abstract

The changes in the cellulose structure of eight *Eucalyptus* species (*E. botryoides*, *E. globulus*, *E. grandis*, *E. maculata*, *E. propinqua*, *E. rudis*, *E. saligna* and *E. viminalis*) in a mild torrefaction (from 160 °C to 230 °C, 3 h) were studied in situ and after cellulose isolation from the wood by solid-state carbon nuclear magnetic resonance (^13^C NMR), wide angle X-ray scattering (WAXS), Fourier transform infrared spectroscopy (FTIR) and by analytic pyrolysis coupled with gas chromatography and mass spectrometry (Py-GC/MS). Changes in molecular weight were assessed by viscosimetry. A small decrease in cellulose crystallinity (ca. 2%–3%) was attributed to its amorphization on crystallite surfaces as a result of acid hydrolysis and free radical reactions resulting in the homolytic splitting of glycosidic bonds. The degree of the cellulose polymerization (DPv) decreased more than twice during the heat treatment of wood. It has been proposed that changes in the supramolecular structure of cellulose and in molecular weight during a heat treatment can be affected by the amount of lignin present in the wood. The limitations of FTIR and Py-GC/MS techniques to distinguish the minor changes in cellulose crystallinity were discussed.

## 1. Introduction

Wood is a natural complex of plant polymers, constituted mainly by cellulose, lignin and hemicelluloses, which provide it with unique physical properties. Wood is the oldest material used by humankind for the most varied of purposes, from elements of household utensils to construction materials. Thus, wood is recognized as an environmentally sound building material, for flooring, furniture, and interior decoration due to its intrinsic esthetic value, excellent physical and mechanical properties, and relatively low price. Although wood has increased its consumer value due to being a sustainable natural resource, some of its shortcomings, such as high hydroscopicity, cause dimensional instability of wood-made products [1]. Another weak point of wood as a material is its biodegradability, i.e., susceptibility to either biotic or abiotic factors [2,3,4], creating some constrains/limitations when applying woody materials outdoors. To overcome these negative aspects, different kinds of protectors (e.g., vanishes, paints, coatings and chemical treatment) have been applied to wood products to enhance its durability [5]. Anaerobic heat treatment has been known since the 1920s as an alternative method of wood protection, providing a radical improvement in the dimensional stability of heat-treated wood [6,7]. Thermal pretreatment or mild torrefaction of wood is usually carried out at moderate temperatures (150–250 °C) to preserve its mechanical properties. Modern industrial approaches for the thermal treatment or heat modification/mild torrefaction, such as, for example, Thermowood^®^ technology, are environmentally friendly and highly effective processes to enhance the dimensional stability and decay resistance of wood without using any toxic chemicals, but they can lower its mechanical properties [8,9]. In this context, the understanding of the thermal behavior of wood components under conditions of thermal pre-treatment of wood is of great relevance.

The composition of the cell wall material in wood and its physical properties are changed by exposure to temperatures greater than 160 °C under oxygen-depleted conditions [10]. It is not entirely clear what actually happens to the structural elements of the wood, and this is a subject of study and discussion. Thus, it is evidenced that hemicelluloses are firstly degraded during thermal treatment of wood by depolymerization via homolytic slitting of glycoside bonds and by hydrolysis, loss of volatile products and extensive deacetylation [8,11,12,13]. Lignin is believed to be more resistant to thermal degradation than polysaccharides [8], but it also undergoes certain condensation and esterification reactions and is partially demethoxylated [14,15]. Recently, the typical lignin reactions of different *Eucalyptus* species thermally treated at 160–230 °C were reported, which included homolytic cleavage of ether linkages responsible for a ca. 25% reduction in its molecular weight and the formation of different condensation structures via radical coupling [16].

Cellulose is the dominant polymer in wood composed of linear chains of β(1→4)-linked β-D-glucopyranose units and forming alternating crystalline and amorphous regions in bulk due to the strong intra- and intermolecular hydrogen bonds [17]. This amorphous-crystalline structure largely affects the cellulose thermal behavior during wood thermal pretreatment. According to common opinion, unlike hemicelluloses, cellulose undergoes much less depolymerization and reveals changes in its physical structure, expressed in the increase in crystallinity due to the partial hydrolysis/recrystallization of the amorphous counterpart [18,19,20,21]. However, as it was widely reviewed [8,9,20], to date, there is no consensus in relation to exact changes in cellulose supramolecular structure under heat treatment. In fact, researchers often report contradictory data about changes in cellulose crystallinity after heat treatment. This can be explained by the diverse thermal treatment conditions used in these studies [22,23,24,25], wood humidity [18], employed equipment [26] and the methods involved to assess the cellulose crystallinity and the fibrils dimensions [22,23,24,25,26,27]. However, it is widely accepted that in addition to hydrolytic reactions due to the release of water and organic acids from wood, the degree of cellulose polymerization is reduced as a consequence of the regeneration of free radicals (homolytic splitting of glycosidic bonds), formation of carbonyl groups and carboxyl, and carbon dioxide [18,19,20,21,26].

Since cellulose is greatly responsible for the mechanical properties of wood, its behavior during the mild torrefaction of wood is of particular interest. The critical aspect is the knowledge on cellulose crystallinity evolution upon heat pretreatment, because the crystalline state significantly influences such properties as the elasticity and absorptive capacity, and other industrially valuable physical properties (strength, density, surface energy, etc.). Accordingly, the aim of this study was to contribute comprehensively to the knowledge on cellulose behavior during thermal pretreatment of wood by employing wide-angle X-ray scattering (WAXS), solid-state carbon nuclear magnetic resonance (^13^C NMR), Fourier transform infrared (FTIR) and pyrolysis, coupled with gas chromatography and mass spectrometry (Py-GC-MS) techniques. For this purpose, the eight *Eucalyptus* species (*E. globulus*, *E. botryoides*, *E. viminalis*, *E. grandis*, *E. rudis*, *E. maculata*, *E. saligna* and *E. propinqua*) submitted to an industrial thermal treatment under controlled conditions (160–230 °C, 3 h) and the treated and non-treated wood samples were evaluated in situ and after the cellulose isolation from some point samples by the aforementioned techniques. This multi-analytical approach allowed a critical examination and new findings of structural changes in cellulose structure during the industrial mild torrefaction of wood.

## 2. Materials and Methods

### 2.1. Raw Materials and Thermal Treatment

Eight *Eucalyptus* wood species were used in this study, all being grown under the same edaphoclimatic conditions in an arboretum localized in the School of Agriculture (ISA), Portugal: *E. globulus*, *E. propinqua*, *E. botryoides*, *E. viminalis*, *E. grandis*, *E. rudis*, *E. maculata*, and *E. saligna*. The trees were harvested at the age of six years, cut in logs and fractionated in boards that were thermo-modified by gradually heating them from 160 to 230 °C during 3 h in an industrial oven provided by Santos & Santos Madeiras, a Portuguese Company under the trademark of Atlanticwood^®^ (http://atlanticwood.pt). After the treatment, the boards were stored under dark at room temperature with 60% relative humidity to stabilize.

### 2.2. Analysis of Wood

Samples of the wood boards were reduced manually to chips, grinded in a cross beater RETSCH mill (SK100) with a 1 mm sieve, and the 40–60 mesh fraction recovered for chemical analysis. The chemical analysis involved the quantification of holocellulose and the sugars’ monomeric composition, where here the focus was on the glucose and acetyl groups, using procedures described in the previous work [28]. The 40–60 mesh fraction of the eight species of wood (before and after thermal treatment) were also analyzed by ^13^C CP/MAS NMR in a Bruker Avance 400 spectrometer (magnetic field of 9.4 T). The samples were spun in a 7 mm zirconia’s rotor (12 kHz), and the following acquisition parameters applied: a proton pulse of ca. 4 µs (90°), contact time of 2 ms, recovery delay of 4 s and 8000–9000 scans accumulated. The Hartman–Hahn matching procedure used glycine that also served as an as external standard for the calibration of the chemical shift scale relative to tetramethylsilane ((CH_3_)_4_Si).

### 2.3. Cellulose Isolation

The cellulose was isolated from the solid residues belonging to *E. globulus* and *E. propinqua*, before and after the heat treatment, using the Kürschner and Hoffer isolation procedure. The residual xylan content did not exceed 3%–4%. Typically, around 1 g of woody material was treated by several consecutive stages with HNO_3_: EtOH mixture (1:4, *v*/*v*) with a liquid-to-solid ratio 50 under reflux for 1 h each. The end of the treatments was checked by Herzberg reagent [29]. The final cellulose residue was washed by ethanol and hot distilled water until neutral reaction of filtrates.

Alternatively, cellulose samples were prepared from peracetic holocellulose obtained by the treatment of wood sawdust (40–60 mesh) with 10% peracetic acid for 20–25 min at 85 °C [30]. The holocellulose was extracted twice with dimethyl sulfoxide (DMSO) at 50 °C for 12 h each extraction to eliminate most of the xylan without damaging the polysaccharides [31]. These cellulose samples were used for the analysis of the degree of polymerization by viscosimetry.

### 2.4. Cellulose Characterization

Isolated cellulose samples (Kürschner and Hoffer) were analyzed as textured samples (ca. 50 mg of cellulose was pressed at 50 MPa in 1 mm thickness pellet of around 1.2 cm diameter) by X-ray diffraction scattering analysis (WADS) in a Philipps X’Pert MPD diffractometer using Cu-Kα source (λ = 0.154 nm) in the 2*θ* range 2°–40° and scanning step width of 0.02°/scan. The background scattering was subtracted from the cellulose diffraction diagram using WINPLOTR software. The amorphous halo was determined after the baseline correction in the 2θ range of 12°–30° assuming a maximum intensity in between 19° and 20°, and the crystalline reflection profiles were fitted using the Lorentzian function. The degree of cellulose crystallinity (*DC*_0_) was calculated from the integral scattering intensities of the crystalline (*I_c_*) and the amorphous (*I_a_*) regions [31] according to Equation (1):

(1) $$DC_{0},\;\%\;=\;\frac{I_{c}}{I_{c}\;+\;I_{a}}\cdot 100$$

The average width of crystallite in the 200-lattice plane (*D*_200_) was determined using the Scherer equation, taking into account the crystallite defects [32]:

(2) $$D_{200},\;nm\;={\left[{\left(\frac{\beta_{200}\cos\;\theta_{200}}{\lambda }\right)}^{2}\;-\;{\left(\frac{\delta_{L}}{d_{L}}\right)}^{2}\right]}^{-\frac{1}{2}}$$

where *β*_200_ is the width on the middle height of the 200 reflection (in rad); *θ_200_* is the maximum of the 200 reflection (in rad); *λ* is the wavelength of the X-ray source (λ = 0.154 nm), *δ_L_* is a parameter related to the lattice distortion perpendicular to the 200 plane direction (0.05), and *d_L_* represents the average distance between the 200 lattice planes (0.395 nm). The dimensions of the cell unit were calculated from Bragg’s equation relating the spacing between the scattered planes, *d*, and the angle between the incident ray and the scattering planes (*θ*, deg.):

(3) $$d=\frac{n\lambda }{2\cdot \sin\;\theta }$$

Fourier transform infrared analysis (FTIR) was performed with the samples in potassium bromide (KBr) pellets and the spectra were acquired on a FT-IR spectrometer (Mattson, Model 7300) at 4 cm^−1^ of resolution, 128 scans per set and over a range of 4000–525 cm^−1^. The crystallinity parameter (*CrP*) was calculated by the ratio of the transmittance peaks recorded at 1372 cm^−1^ (T_1372_) and at 2900 cm^−1^ (T_2900_) according to Tripp [33], as presented in Equation (4):

(4) $$CrP,\;\%\;=\;\frac{T_{1372}}{T_{2900}}\cdot 100$$

Other indices were also calculated based on the absorbance values (A = 2-log(T)). The lateral order index (LOI) was calculated as the ratio between the absorbance at 1423 cm^−1^ (associated with the amount of crystalline cellulose) and the absorbance at 897 cm^−1^ (assigned with the glycosidic bond β-(1,4) stretching in cellulose). The HBI (hydrogen bond intensity) was obtained as the ratio from absorbance at 3336 cm^−1^ (OH stretching, H-bonds between molecules) and the absorbance at 1335 cm^−1^ (CH rocking vibrations of the glucose ring) [34].

Cellulose was also analyzed by ^13^C CP-MAS NMR (solid-state Cross Polarization—Magic Angle Spinning Nuclear Magnetic Resonance). The spectra were recorded on a Bruker Avance 400 spectrometer, where the samples were packed into a zirconia’s rotor sealed with Kel-F^TM^ caps and spun at 12 kHz. The acquisition parameters were the following: ca. 7000 scans with a 90° proton pulse, a 1 ms of cross-polarization contact time and a 2.5 s of recovery delay.

The crystallinity index (*CrI*) was calculated from the relationship between the integration areas of the ordered (A_86–92_ ppm) and amorphous (A_79–86_ ppm) C-4 signals in cellulose [35] using the Equation (5).

(5) $$CrI,\;\%\;=\;\frac{A_{86-92\;ppm}}{A_{86-92\;ppm}+A_{79-986\;ppm}}\cdot 100$$

The index for the carboxyl/ester groups (I_CO2_) was calculated based on the carbon signals from the corresponding groups at 165–178 ppm using the intensity of anomeric carbon (C1) in cellulose at ca. 105 ppm as an internal standard. The lateral dimensions of fibril aggregates were calculated as described by methodology proposed by Wickholm et al. [36] according to the model of a fibril with a square cross-section given by the equation:

(6) $$q=\frac{4n-4}{n^{2}}$$

where *q* is the fraction of intensity of the signal of accessible surfaces. The accessible and inaccessible cellulose surfaces were estimated by deconvolution of the corresponding signals in ^13^C CP-MAS NMR spectra (Figure S1 in Supplementary Materials). The calculated number of cellulose chains perpendicular to the fibril cross-section along one side of the assumed square fibril or the assumed square fibril aggregate cross-section *n* can be converted to a lateral dimension expressed in nm using a factor of 0.55 nm per chain.

The cellulose samples were analyzed by analytic pyrolysis coupled with gas chromatography and mass spectrometry (Py-GC/MS) after milling in a Retsch MM20 mixer ball mill (10 min). Around 0.10 mg of cellulose was pyrolyzed (550 °C for 1 min) in a platinum coil Pyroprobe connected to a CDS 5150 valved interface linked to a gas chromatographer (Agilent 7890B) with a mass detector (5977B), and using a fused-silica capillary column (ZB-1701: 60 m × 0.25 mm i.d. × 0.25 µm film thickness). The chromatographic conditions used were: 40 °C, held for 4 min, 10 °C min^−1^ to 70 °C, 5 °C min^−1^ to 100 °C, 3 °C min^−1^ to 265 °C, held for 3 min, 5 °C min^−1^ to 270 °C, held for 9 min. The temperatures applied were as follows: 270 °C (injector), 280 °C (MS interface). The electron ionization energy was at 70 eV. Helium was the carrier gas with a total flow of 1 mL/min. The compounds were identified comparing their mass spectra with Wiley, the NIST2014 database and the literature [37,38]. The total area of the chromatogram was obtained automatically and the percentage area of each compound identified calculated.

The average viscosimetric degree of the polymerization (DPv) of cellulose was calculated from the Equation (6), proposed by Evans and Wallis [39] and using the data for the intrinsic viscosity ([η]) obtained according to SCAN-CM 15:88 in cupriethylenediamine solution:

(7) $$1.1\left[\eta \right]={\left(DPv\right)}^{0.85}$$

## 3. Results and Discussion

### 3.1. Chemical Analysis of the Thermally Treated Woods

A summary of the chemical analyses of the eight *Eucalyptus* species showed that the heat treatment promotes an average mass loss of ca. 11% among the woods, with the major loss being observed for the *E. propinqua*, where the loss was as higher as 12%, followed by *E. viminalis* and *E. saligna* (Table 1). *E. maculata*, *E. globulus* and *E. grandis* showed slightly lower mass loss (ca. 10%). Holocellulose content showed an average reduction of 16%, while in *E. propinqua* and *E. globulus*, the content decreased by 19% and 16%, respectively. This reduction was mostly due to the degradation of xylan since the percentage of glucose (based on total sugars) increased more or less equally in all species in the order of 20% (*E. propinqua*) to 33% (*E. viminalis*) (Table 1). In addition, the drastic reduction in acetyl groups belonging to xylan was evident in all treated woods (average of 57%), in percentages ranging from 40% (*E. propinqua*) to 56% (*E. rudis*). The opposite trend was observed in the total lignin content, which increased by an average of 33%, where *E. maculata* showed the highest growth (60%) and *E. viminalis* the lowest (23%). The total lignin content accounts for the lignin itself and some other concomitants (*e.g*., tannins) and heat degradation products resulting from carbohydrates and extractives [16]. These results are coherent with others previously reported for the hardwoods’ thermal treatment at a similar range of temperatures [26,40,41] and evidenced the relative stability of lignin and cellulose towards thermal destruction.

It is noteworthy that the bond dissociation energy (BDE) of glycoside linkage in cellulose [42] and of ether linkages in lignin [43] are of the same order (ca. 54 and 40–60 kcal.mol^−1^, respectively), but the glycoside bonds are much more susceptible to hydrolysis that the ether bonds in lignin. That is why the main degradation of the cellulosic chains is attributed mainly to the hydrolysis of accessible cellulose regions, rather than to homolysis, which becomes quite significant at temperatures as high as 240–250 °C [22]. On the other hand, the high cellulose crystallinity and the arrangement of cellulose fibrils in the cell wall (fibrils are embedded to the lignin matrix) drastically decrease the cellulose ability to undergo hydrolysis catalyzed by the organic acids released in the heat treatment of wood. It is proposed that lignin could play a role of cellulose protector in the heat treatment of wood [44].

Regarding the structural changes of cellulose in the mild torrefaction of wood (160–240 °C), there are strong arguments in favor of both a possible increase and decrease in the degree of cellulose crystallinity (DC). On the one hand, the amorphous cellulose is more susceptible to hydrolysis induced by wood degradation products than the crystalline regions. This could naturally increase the crystalline cellulose phase, also due to the partial recrystallization of the amorphous and paracrystalline regions and cocrystallization of neighboring crystallites [18,19,20,21,22,23]. On the other hand, part of the crystalline cellulose can be also amorphisized from the fibrils’ surface due to the same hydrolysis reactions, homolytic splitting of glycoside bonds and the cellulose chains’ rearrangements [23]. The latest transformations are certainly prevalent at temperatures as high as 240–250 °C [22,23]. However, at temperatures below 240 °C, both a decrease [25,45] and an increase [24] in cellulose DC have been reported. This point was of particular attention in this study and evaluated by a set of analytical techniques recognized in the area. 

### 3.2. Changes in Cellulose Structure during the Thermal Treatment

#### 3.2.1. CP-MAS ^13^C NMR Analysis

The structural features of cellulose during the thermal treatment of different *Eucalyptus* species were analyzed by solid-state ^13^C NMR using integral wood sawdust. Semi-quantitative analysis was carried out using the crystallinity index (*CrI*) of cellulose and estimating the amounts of carboxyl and ester groups (–CO_2_−) per one glucopyranose unit using the signal of C1 in cellulose as an internal standard (Table 2). The amount of the carbonyl groups was too small and they were not considered for the analysis. The *CrI* relates to cellulose DC reflecting the relative proportion of conventional crystalline and amorphous cellulose [35]. *CrI* is always smaller than DC and is a useful indicator for comparative reasons.

**Figure 1:**
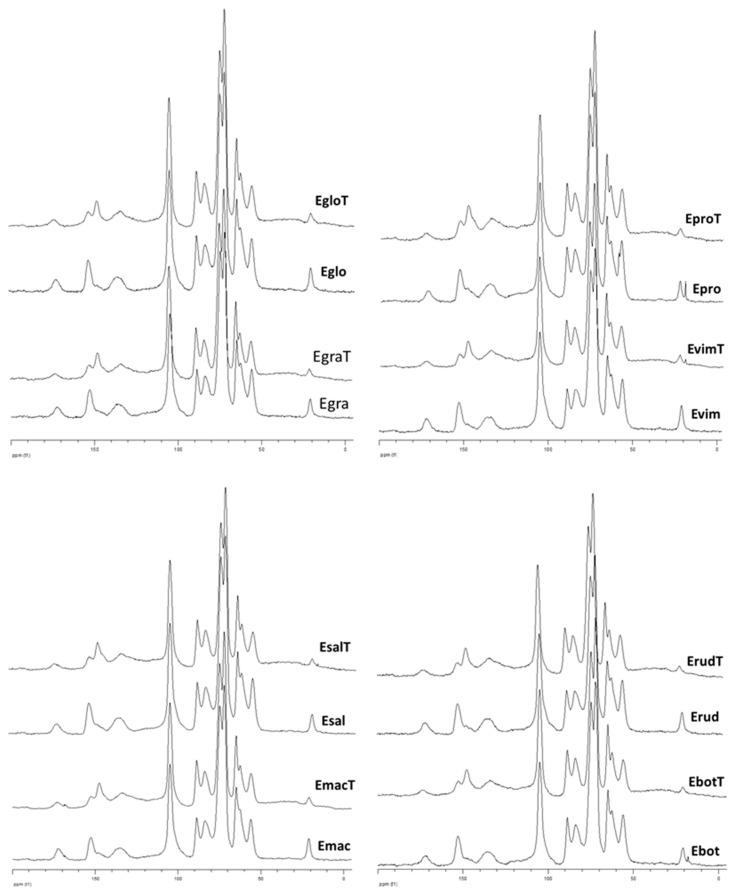
^13^C CP/MAS NMR spectra of the *Eucalyptus* woods before and after thermal treatment: *E. globulus* (Eglo/EgloT), *E. propinqua* (Epro/EproT), *E. grandis* (Egra/EgraT), *E. viminalis* (Evim/EvimT), *E. saligna* (Esal/EsalT), *E. rudis* (Erud/ErudT), *E. maculata* (Emac/EmacT), *E. botryoides* (Ebo/EboT).

The *Eucalyptus* species showed similar spectral features before and after the thermal treatment (Figure 1). The *CrI* was always greater for the treated than for the untreated woods. However, this fact does not indicate an unconditional increase in the DC of cellulose during the thermal treatment of wood. The fact is that the spectra regions used for the *CrI* determination belong not only to cellulose, but also to lignin and hemicelluloses (mainly xylan). The spectrum region at 79–86 ppm is especially critical, because it contributes simultaneously to the C4 in glucopyranose units of predominantly amorphous cellulose, to C_β_ in β-*O*-4 lignin structures and to C3 of the xylan [35]. Independently of the cellulose structural behavior, the integral intensity of this spectrum region in a treated wood decreases due the appreciable cleavage of β-*O*-4 lignin structures [16] and a drastic degradation of the xylan (Table 1). Therefore, the inferred *CrI* of cellulose is significantly overestimated and does not reflect unambiguously the changes in the supramolecular structure of the cellulose. The adequate deconvolution and subtraction of signals other than those of cellulose from the aforementioned region is very difficult due to the uncertainty of the contribution of each of them.

The profile of carboxyl/ester groups in treated/non treated woods was ambiguous, showing simultaneously the same (*E. propinqua* and *E. saligna*), greater (*E. globulus*) or lower (*E. botryoides*, *E. viminalis*, *E. grandis*, *E. rudis* and *E. maculata*) levels after the treatment (Table 2). Again, these –CO_2_–groups belong to cellulose, lignin and hemicelluloses, which have different behavior under conditions of heat treatment. Supposing the formation of new carboxyl/ester groups in cellulose and lignin during the treatment, the strong deacetylation of the xylan might be taken into account. In fact, a notable loss of acetyl groups from xylan in a range from 18% (*E. grandis*) to 46% (*E. saligna*) was detected (Table 2), which negatively contributed to the intensity of signals at 165–178 ppm. Hence, the clear tendency towards the formation of new oxidized groups in cellulose during the heat treatment was inconclusive. 

**Figure 2:**
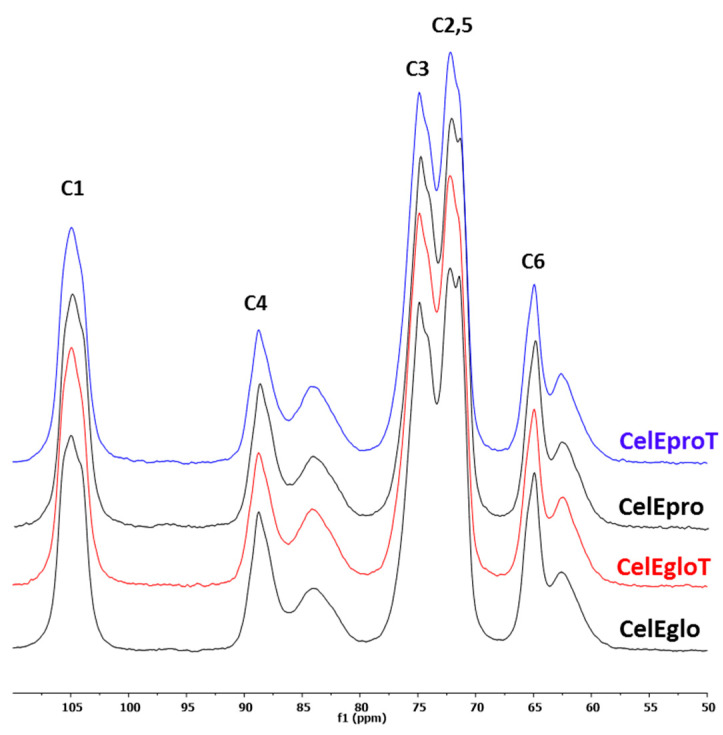
^13^C CP/MAS NMR spectra of cellulose samples isolated from *Eucalyptus* woods before (Cel**Eglo** and Cel**Epro**) and after (Cel**EgloT** and Cel**EproT**) thermal treatment.

In order to assess more accurately the structural changes in cellulose during the thermal treatment, cellulose was isolated from the wood and analyzed again by solid-state ^13^C NMR (Figure 2). The Kürschner and Hoffer method, used in this study for the cellulose isolation, is usually employed for the quantitative cellulose analysis in plant materials and does not significantly change the physical structure of the cellulose [31]. Two eucalypt species were selected for comparative reasons, containing the lowest (*E. globulus*) and the highest (*E. propinqua*) lignin content (Table 1).

The results on *CrI* analysis clearly showed the decrease in cellulose crystallinity in both wood species during the thermal treatment (Table 3). In addition, the lateral dimensions of cellulose fibril aggregates (*A*) were reduced in the treated wood. The last fact indicates that the cellulose fibril surfaces in the treated wood contain some amorphous phase or non-cellulosic compounds (e.g., hemicellulose) that hindered the aggregation of the fibrils upon drying. The fibrils’ aggregation is strongly dependent of their surface purity and decreases drastically in the presence of hemicelluloses [35,46]. The only fibrils free of other concomitant cell wall polymers (*e.g*., hemicelluloses and lignin) are susceptible to the cocrystallization through the coalescence of neighboring crystallite regions with a small increase in DC [46,47]. However, the isolated cellulose samples did not contain lignin (negative reaction on the Herzberg reagent) and contained minimal amounts of hemicelluloses (less than 3%). Therefore, the surface amorphization of cellulose in fibrils during the heat treatment could be proposed.

The analysis of the ^13^C CP/MAS NMR of cellulose also showed some changes in the relative proportion of cellulose phases I_α_ and I_β_ (Figure S1 in Supplementary Materials) after the wood treatment. The relative intensity of the C1 signals at 104 and 106 ppm decreases slightly and the signal at 105 ppm increases in the cellulose from the treated wood (Figure 2). The C1 signals at 104 and 106 are assigned to cellulose I_β_ and the signal at 105 ppm is essentially assigned to the cellulose I_α_ [48]. The cellulose phase I_α_ has the triclinic unit cell and is located on the surface of the crystallite along the elementary fibrils, being alternated with phase I_β_ that has the monoclinic unit cell [49,50]. Since the I_β_ phase is dominant in plant celluloses, it might constitute the major core of crystallites. The phase I_α_ is metastable and usually converted to I_β_ by annealing [49]. Therefore, the eventual assumption on the I_β_ → I_α_ transformation to explain the I_β_ phase reduction would be untenable. The plausible explanation could be the eventual partial amorphization of cellulose chains under heat treatment on the surface of crystallites that were removed, at least partially, jointly with hemicelluloses during the isolation of Kürschner and Hoffer cellulose under strong acidic conditions. In this case, the effective width of the crystallite could be reduced, leading to less phase I_β_, based on the proposition that the surface coverage with phase I_α_ remains similar. Previously, phase I_β_ reduction has also been reported in heat treated wood during the analysis of cellulose isolated by the same method [25].

The moderate depolymerization of cellulose during the heat treatment of wood was confirmed when comparing DPv of isolated cellulose before and after the treatment (Table 3). The cellulose samples used in these analyses were obtained from corresponding peracetic holocelluloses that were thoroughly extracted by DMSO. Although these cellulose samples still contained hemicelluloses, they did not degrade as much as isolated α-cellulose samples explored previously for the same purposes [26]. This can explain some greater DPv values of cellulose obtained in our study when compared to those reported for the same wood and determined from the α-cellulose samples [26].

#### 3.2.2. X-ray Scattering Analysis (WAXS)

**Figure 3:**
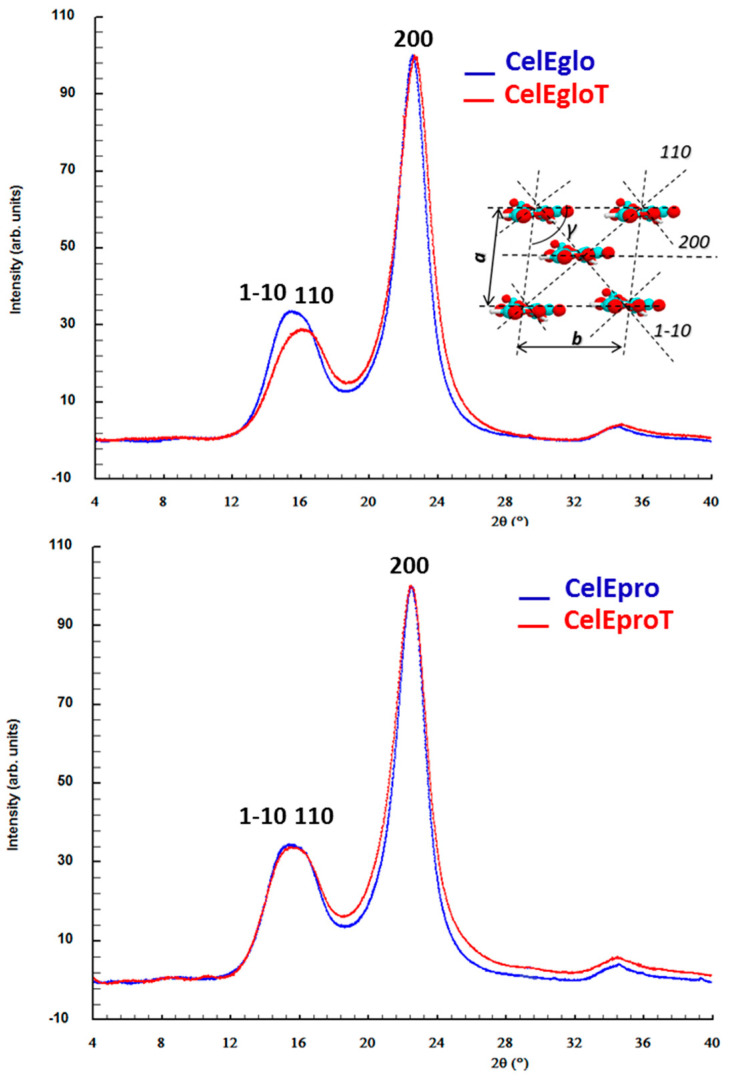
X-ray scattering diffractograms of cellulose isolated from two eucalypt species before (CelEglo and CelEpro) and after (CelEgloT and CelEproT) the heat treatment. The schematic representation shows the crystalline unit cell and the main lattice plans.

Since the accurate determination of the DC of cellulose in wood by X-ray scattering is a complex analytical problem [32], these analyses were carried out using cellulose samples isolated from wood by the method of Kürschner and Hoffer. The WAXS analysis revealed changes in the diffractograms of cellulose samples isolated from untreated and thermally modified wood, dealing with some enlargement and a small change in the maximum reflections (Figure 3). This reveals changes in the lateral dimensions of crystallites (D_200_) and in the parameters of the elementary unit cell of cellulose I (Table 3). The obtained results clearly indicated the decrease in the DC of cellulose (ca. 2.5%) after heat treatment for both examined species (*E. globulus* and *E. propinqua*). This is in tune with results obtained by solid-state ^13^C NMR (Table 2). At the same time, WAXS data showed a decrease in D_200_ after the wood treatment. Hence, everything indicates a partial amorphization of cellulose on the crystallite surface during the mild torrefaction of the wood. The certain decrease in cellulose DC and/or the reduction in the average lateral dimension of cellulose crystallites under similar conditions of heat treatment used here were reported previously by other researchers both for hardwoods and softwoods [23,40,44].

It is noteworthy that greater changes in the DC and D_200_ of cellulose during heat treatment were observed for *E. globulus* than for *E. propinqua* (Table 4). This is coherent with data on the CrI obtained by solid-state ^13^C NMR (Table 3). Such a singularity may be related to the notably higher lignin content in *E. propinqua* when compared to *E. globulus*. The higher coverage of cellulose fibrils with lignin could favor better fibrils protection against acid-catalyzed degradation and the cleavage of glycoside bonds through free radical mechanisms during the heat treatment of wood.

#### 3.2.3. FTIR Analysis

**Figure 4:**
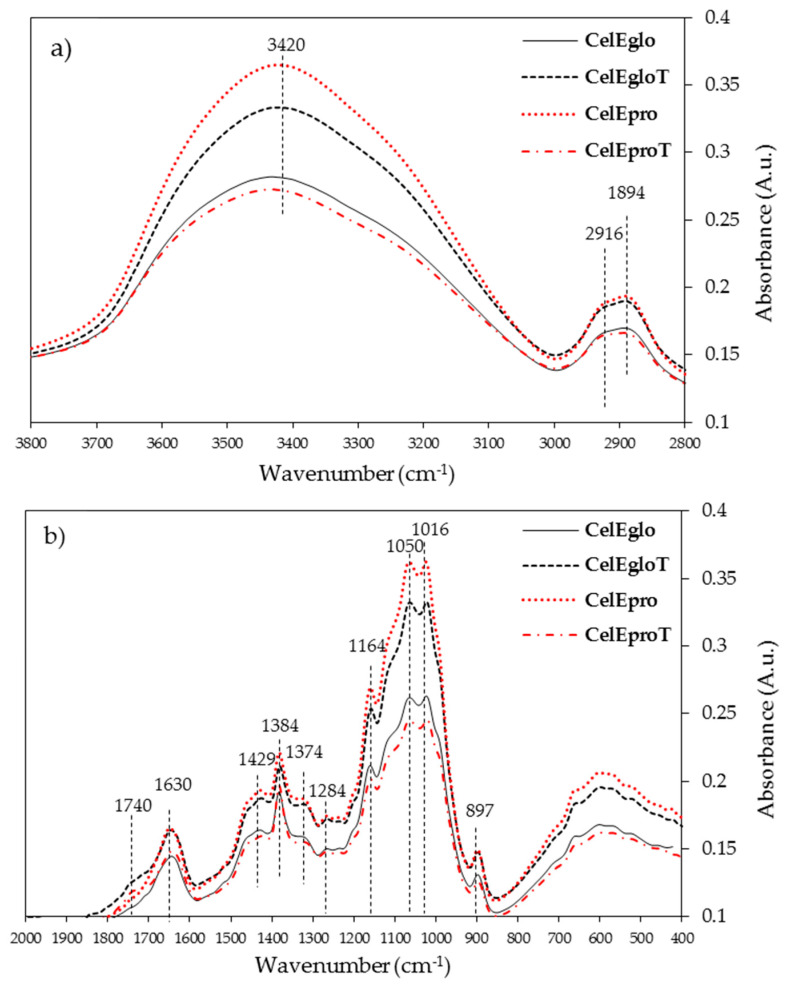
FTIR spectra of the isolated celluloses depicted in the region 3800–2800 cm^−1^ (**a**) and in the region 1800–600 cm^−1^ (**b**). The samples designations are the same as in Table 2 and Table 3.

Figure 4 shows the FTIR spectra of the isolated celluloses that are depicted in two regions: the 2800–3800 cm^−1^ (Figure 4a) and the 600–1800 cm^−1^ (Figure 4b) region. The celluloses from untreated woods were quite different from each other; in **CelEpro** the band between the 3600 and 3100 cm^−1^ region is sharper and with higher intensity comparative to **CelEglo**, and the opposite features occurred in the cellulose samples from treated woods. The band between the 3600 and 3100 cm^−1^ region is related to the O-H stretching, but also to the intensity of intramolecular and intermolecular hydrogen bonding [33,51]. The decrease in intensity of this band is related to the scission of intra- and intermolecular hydrogen bonds, meaning that these linkages were weaker in **CelEglo** than in **CelEgloT** and in **CelEproT** than in **CelEpro**. In addition, the amount of hydroxyl groups, absorbed water and the hydrogen bonding were more pronounced in the cellulose from *E. propinqua* (**CelEpro**) and *E. globulus* after the treatment (**CelEgloT**) due to the higher intensity of this band in these samples, which is in accordance with the higher glucan content in *E. propinqua* wood and in the *E. globulus* after the treatment (Table 1). The band centered at ca. 2900 cm^−1^ consists of overlapped bands at 1894 and 2916 cm^−1^ assigned to antisymmetric and symmetric CH_2_ stretching, respectively [33]. These bands followed the same intensity features as the bands centered at ca. 3420 cm^−1^ and were used for the evaluation of cellulose crystallinity as the reference.

The “fingerprint” region (1500 to 800 cm^−1^) presents similar signal profiles for all cellulose samples, showing, however, some differences in the intensity of the bands (Figure 4b). The absence of characteristics for lignin bands at 1737, 1594, 1510, and 1263 cm^−1^ and the strong band characteristics for the xylans at 1730–1740 cm^−1^ are noteworthy, thus evidencing the purity of isolated cellulose samples.

Table 5 summarises the values of CrP (crystallinity parameter), TCI (total crystallinity index), LOI (lateral order index) and HBI (hydrogen bond intensity). The CrP refers to the abundance of crystalline cellulose contributing substantially to the band at 1372 cm^−1^ assigned to C-H bending [33]. This parameter slightly decreased in *E. globulus* cellulose and increased in *E. propinqua* cellulose isolated after the wood heat treatment. The TCI represents the total crystallinity index, similar to the physical meaning of CrP, and the values were quite similar between cellulose samples isolated from untreated and treated wood samples (Table 5). The values for the empirical crystallinity index (lateral order index, LOI) are practically the same for cellulose from *E. globulus* and *E. propinqua* wood and unchanged after the thermal modification of wood (Table 5). The band at 1430 cm^−1^ refers to asymmetric CH_2_ bending vibration in the crystalline phase and the band 897 cm^−1^ is specific for the CH deformation vibrations in the glucopyranose ring [51].

Since the band at 3336 cm^−1^ is assigned to O-H stretching and hydrogen bonding between molecules and the band at 1335 cm^−1^ is assigned to CH rocking vibrations of the glucose ring, their ratio, known as the hydrogen bond intensity (HBI), relates to the degree of intermolecular regularity and the amount of bound water [34,51]. The HBI values in cellulose from untreated wood show that *E. propinqua* has a stronger hydrogen bonding when compared to *E. globulus* (1.95 vs. 1.77), but the heat treatment breaks more of these bonds in the case of *E. propinqua*, because the HBI decreased to 1.76. At the same time, these bonds were enhanced by heat treatment in the case of *E. globulus* since the value increased from 1.77 to 1.82. It seems that the FTIR technique is less sensitive in distinguishing the minor changes in cellulose crystallinity when compared to solid-state ^13^C NMR and WAXS. This was probably the reason why coherent conclusions were drawn for the structural changes of cellulose during the heat treatment of *E. globulus* wood by all these techniques and less conclusive results were obtained for *E. propinqua*, whose cellulose crystallinity changed less with the wood treatment (Table 3 and Table 4).

#### 3.2.4. Pyrolysis Analysis

Py-GC/MS is a recognised powerful technique for the study of the chemical compositions of complex polymeric systems, including wood. The pyrolysis products reflect not only the structural composition of polymer composites, but also their physical structure. Regarding the major wood components, lignin and carbohydrates, they give completely different pyrolytic degradation products, the relative proportions of which allow the discrimination of a wood’s botanic origin and the assessment of its behaviour during different processing routes [52]. In particular, the main pyrolysis product of cellulose (60%–70% yield) is levoglucosan (LG), followed by low molecular compounds (LMC) and furan-derived products, contributing by 10–20% and by 5–15%, respectively [52,53,54,55]. LG is a pyran-derived product from cellulose pyrolysis and is formed by cleavage of the 1,4-glycosidic linkages, followed by intramolecular rearrangement of the monomer unit and dehydration. Under the same pyrolysis conditions, the yield of LG is related to the physical properties of cellulose and is usually higher for the amorphous than for the crystalline phase [53,54,55]. However, there is not yet a full consensus regarding the influence of the degree of polymerization and the crystallinity of cellulose upon the formation of LG [56].

**Figure 5:**
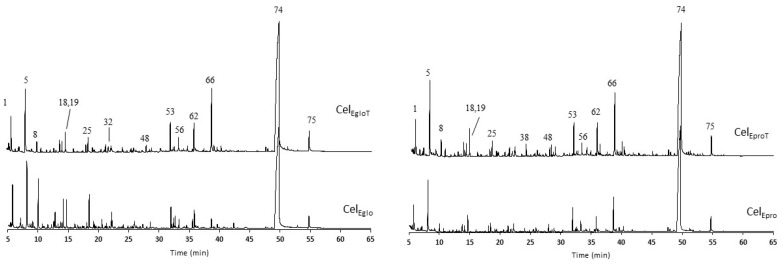
Pyrograms of the celluloses from *E. globulus* and *E. propinqua* before (CelEglo, CelEpro) and after the thermal treatment (CelEgloT, CelEproT). Peak identification: 1—2-oxo-propanal; 5—hydroxyacetaldehyde; 8—acetol; 18—furfural; 19—2-cyclopenten-1-one; 25—2-hydroxy-2-cyclopenten-1-one; 32—2H-pyran-2-one; 38—guaiacol; 48—levoglucosenone; 53—NI sugar; 56—1,4:3,6-dianhydro-α-D-glucopyranose; 62—5-hydroxymethylfurfural; 66—2-hydroxymethyl-5-hydroxy-2,3-dihydro-(4H)-pyran-4-one; 74—1,6-anhydro-β-D-glucopyranose (levoglucosan); 75—1,6-anhydro-α-D-galactofuranose. More peak assignment is provided in the supplementary information.

The results from the Py-GC/MS of the isolated celluloses and the corresponding pyrograms are presented in Table 6 and Figure 5, respectively. The detailed list of pyrolysis products is presented in Table S1 (Supporting Materials). In general, the isolated cellulose samples from heat treated woods produced less carbohydrate derivatives: ca. 84 vs. 87% for the *E. globulus* and ca. 71% vs. 85% for the *E. propinqua*. Since the isolated cellulose samples contained minimal amounts of hemicelluloses, these features can be essentially assigned to the crystalline and amorphous phases of cellulose. Thus, the results are presented by grouping the pyrolysis products in pyran, furan, LMC and others instead of focusing on their origin. The pyrolysis of samples mainly revealed the production of pyran compounds (48–63%), followed by LMC (9–17%), and the furan compounds were less than 7% of the total chromatographic area (Table 6). The main pyran compound was levoglucosan (LG, peak 74), while hydroxyacetaldehyde (peak 5) was predominant among LMC, and the 5-hydroxymethylfurfural (5-HMF, peak 62) was the most abundant furan compound (Figure 5). The Py-GC/MS results indicated that the heat treatment of *E. globulus* wood decreases the molecular ordening of cellulose, because the yield of LG was reduced substantially. This confirms the previous results obtained by solid-state ^13^C NMR, WAXS and FTIR. At the same time, the same conclusion cannot be drawn for the *E. propinqua* wood, where the LG yield decreased in cellulose isolated from treated wood (Table 6). However, the amount of furan-derived pyrolysis products was increased anyway after the treatment of *E. propinqua* wood. The increase in furans and LMS during the pyrolysis of cellulose is clearly indicative of the greater content of amorphized cellulose [55].

The results of the Py-GC/MS analysis on the LG yield can be affected by the concomitant lignin in the isolated cellulose samples. In fact, the analysis of pyrograms showed trace amounts of lignin-derived products in the **CelEglo**, **CelEgloT** and **CelEpro** samples (Table 6). However, these were almost ten times more abundant in the **CelEproT** sample, indicating a much more relevant lignin content. This observation is consistent with a higher Klason lignin content in the *E. propinqua* when compared to *E. globulus* (29.4 vs. 21.1% in untreated and 39.0 vs. 29.1% in the treated wood, Table 1). In addition, despite the predominace in syringyl structural units (S units), the *E. propinqua* is richer than *E. globulus* in guaiacyl lignin units (G units) with an S/G ratio of 1.3 vs. 4.1 and 1.7 vs. 4.6, respectively, in untreated and treated wood [16]. Accordingly, *E. propinqua* wood is much more difficult to delignify, and during the cellulose isolation some lignin was irremovable and mantained in the cell matrix. This fact explains, at least partially, the much lower yield of cellulose-derived pyrolysis products from *E. propinqua* than from *E. globulus*, due to the higher chair yield in the presence of lignin (Table 6).

It can also be speculated that the residual lignin in **CelEproT** affected the results of the FTIR, thus contributing to the signals at 1300–1500 cm^−1^ used to evaluate the cellulose crystallinity.

## 4. Conclusions

The results of this study showed a small decrease in the crystallinity of cellulose (ca. 2–3%) in eucalyptus woods, during the mild torrefaction performed in the range of 160–230 °C (3 h), within the trend of Thermowood^®^ technology. This was attributed to the cellulose amorphization of crystallite surfaces as a result of acid-catalyzed hydrolysis and free radical reactions from the homolytic spitting of glycosidic bonds. The parameters of the crystalline cell unit changed insignificantly under conditions of heat treatment, showing its minor distortion within the allomorph of cellulose I. The degree of the cellulose polymerization decreased more than twice during the heat treatment of wood. It has been proposed that changes in the supramolecular structure of cellulose and in molecular weight during a heat treatment can be affected by the amount of lignin present in the wood. Unlike hemicelluloses, lignin is much less depolymerized with minimal release of low molecular weight products. The increase in the content of lignin and other polyphenols protects the wood cellulose against degradation, decreasing its accessibility for hydrolysis and free radical reactions by radical scavenging. Among the analytic techniques employed, solid-state ^13^C NMR and WAXS are considered more sensitive than FTIR and Py-GC/MS for the unequivocal assessment of small changes in cellulose crystallinity.

## Figures and Tables

**Table 1:** Chemical composition (%, oven dry material) of eucalypt woods before (NT) and after (T) the thermal treatment *.

Species		Weight Loss (%)	Total Lignin (%)	Holocellulose (%)	Glucose (%)	Xylose (%)	Acetyl Groups (%)
*E. globulus*	NT	-	23.9	66.2	68.1	20.6	7.1
	T	9.7	30.0	55.6	82.2	11.8	4.1
*E. propinqua*	NT	-	31.5	63.6	69.2	19.6	7.0
	T	12.7	39.8	51.1	82.7	10.6	4.2
*E. botryoides*	NT	-	29.9	67.5	70.2	19.1	7.0
	T	10.2	37.6	51.1	85.1	8.8	4.0
*E. viminalis*	NT	-	28.4	61.4	59.2	27.0	9.4
	T	12.5	34.9	51.9	81.0	11.2	4.5
*E. grandis*	NT	-	26.0	60.4	69.1	19.5	7.5
	T	9.7	34.8	54.0	85.1	8.8	4.0
*E. rudis*	NT	-	27.6	57.6	64.9	22.5	8.5
	T	11.4	38.1	51.5	85.4	8.8	3.7
*E. maculata*	NT	-	22.5	65.6	61.7	26.4	8.0
	T	9.6	36.1	52.5	82.0	10.5	4.6
*E. saligna*	NT	-	28.3	63.5	70.6	18.3	7.2
	T	12.4	36.8	52.8	84.8	9.1	4.2

* The glucose, xylose and acetyl groups content were reported as percentage of total sugars.

**Table 2:** Analysis of the woods before (NT) and after the treatment (T) by solid-state carbon nuclear magnetic resonance (^13^C NMR).

Species		*CrI* *	I_CO2_ **
*E. globulus*	NT	0.37	0.08
	T	0.44	0.09
*E. propinqua*	NT	0.38	0.12
	T	0.43	0.12
*E. botryoides*	NT	0.38	0.10
	T	0.44	0.09
*E. viminalis*	NT	0.39	0.16
	T	0.44	0.06
*E. grandis*	NT	0.38	0.11
	T	0.43	0.04
*E. rudis*	NT	0.35	0.14
	T	0.43	0.13
*E. maculata*	NT	0.36	0.16
	T	0.46	0.11
*E. saligna*	NT	0.42	0.14
	T	0.45	0.14

* *CrI*—index of cellulose crystallinity. ** calculated as the integral ratio at 165–178 and 100–108 ppm.

**Table 3:** Crystallinity of the isolated cellulose from *Eucalyptus* woods assessed by solid-state ^13^C NMR, degree of polymerization (DPv) determined by viscosimetry.

	CelEglo	CelEgloT	CelEpro	CelEproT
**A** (±0.3 nm)	12.5	9.9	11.5	9.6
**CrI** (±1%)	58	52	57	53
**DPv** (±20)	1300	530	1330	590

**Table 4:** Crystallinity, lateral dimension of crystallites and the crystallite cell parameters of cellulose assessed by wide angle X-ray scattering (WAXS).

	CelEglo	CelEgloT	CelEpro	CelEproT
**DC** (±0.5%)	71.3	68.8	71.6	69.8
**D200** (±0.1 nm)	5.4	4.6	5.5	4.9
**a** (±0.002 nm)	0.797	0.788	0.798	0.795
**b** (±0.002 nm)	0.825	0.835	0.828	0.832
**γ** (±0.1 deg.)	97.0	96.1	97.2	96.5

**Table 5:** Values of the indexes calculated from FTIR spectra.

	CelEglo	CelEgloT	CelEpro	CelEproT
**CrP** (T_1372_/T_2892_ × 100%)	97.9	97.2	96.0	97.2
**TCI** (A_1375_/A_2900_)	1.10	1.10	1.13	1.12
**LOI** (A_1429_/A_897_)	1.25	1.27	1.29	1.27
**HBI** (A_3336_/A_1335_)	1.77	1.82	1.95	1.76

CrP—Crystallinity parameter (%); TCI—total crystallinity index; LOI—lateral order index; HBI—hydrogen bond intensity.

**Table 6:** Pyrolysis results of the isolated celluloses from *E. globulus*, *E. propinqua* before and after thermal treatment (% of total chromatographic area).

	CelEglo	CelEgloT	CelEpro	CelEproT
**Total carbohydrates** (% of total area)	87.1	84.2	84.5	71.1
**Furan**	6.7	5.1	5.2	6.0
**Pyran**	53.6	63.3	60.8	47.6
**Low molecular compounds (LMC)**	16.6	8.3	9.4	9.4
**Others**	10.2	7.5	9.1	8.0
**Total lignin** (% of total area)	0.1	0.1	0.5	3.3
**% of identified compounds**	87.2	84.3	85.0	74.3

## Supplementary Materials

The following information is available online at https://www.mdpi.com/2073-4360/12/12/2831/s1: Figure S1 (^13^C CP/MAS NMR spectrum of CelEglo showing the expanded region at 80–92 and 100–110 ppm with deconvolution of signals from accessible (AC) and inaccessible (IN) cellulose and from cellulose I_α_ and I_β_ phases) and Table S1 (Detailed list of compounds attained by Py-GC/MS analysis).
