# Supplementary material for: Cellulose Structural Changes during Mild Torrefaction of Eucalyptus Wood
Source: Polymers (Basel). 2020 Nov 28;12(12):2831. doi: 10.3390/polym12122831 (PMC7760041; doi:10.3390/polym12122831)
Supplement: Supplementary file 1 [file polymers-12-02831-s001.pdf]

polymers

Supplementary Materials

Cellulose Structural Changes During Mild Torrefaction of *Eucalyptus* Wood

Ana Lourenço [1]*, Solange Araújo [1], Jorge Gominho [1], Dmitry Evtuguin [2]*

[1] Forest Research Center, School of Agriculture, University of Lisbon, Tapada da Ajuda 1349-017 Lisboa; analourenco@isa.ulisboa.pt (A.L); araujo@isa.ulisboa.pt (S.A.); jgominho@isa.ulisboa.pt (J.G.);

[2] CICECO, Chemistry Department, University of Aveiro, Campus de Santiago, P-3810-193 Aveiro; dmitrye@ua.pt

* Correspondence: analourenco@isa.ulisboa.pt; Tel.:+351 213653384 (A.L.); dmitrye@ua.pt; Tel.:+351 234401526 (D.E.)

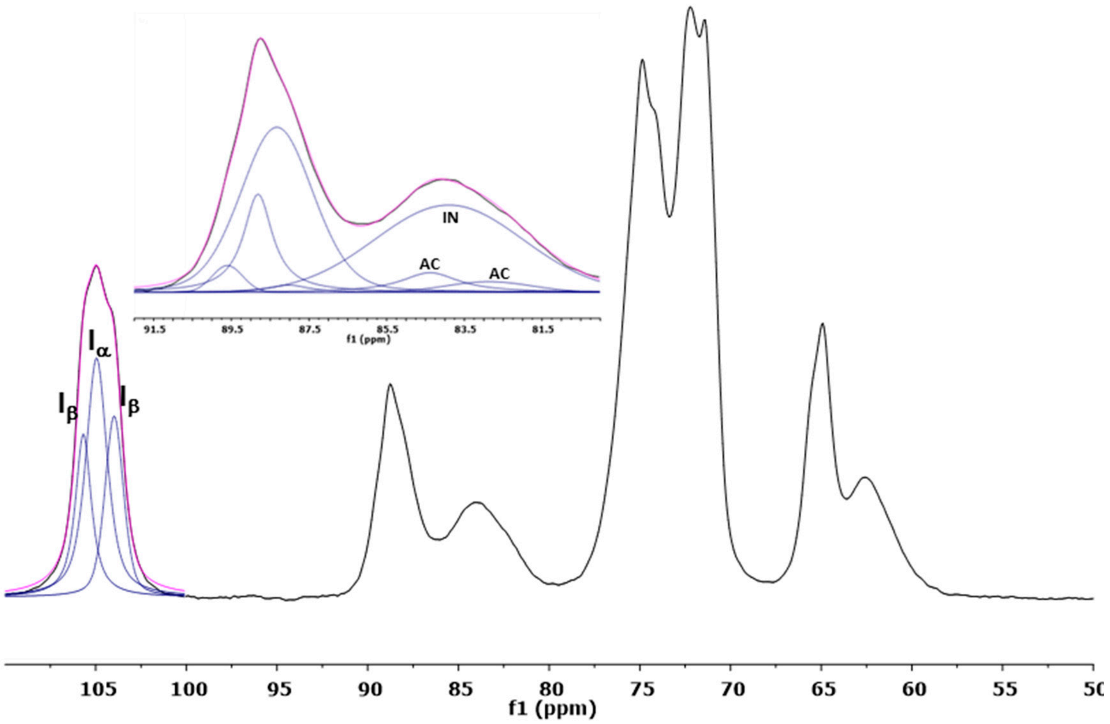

Figure S1. ^{13}C CP/MAS NMR spectrum of cellulose sample isolated from *E. globulus* (**CelEglo**) showing the expanded region at 80-92 and 100-110 ppm with deconvolution of signals from accessible (AC) and inaccessible (IN) cellulose and from cellulose I_{α} and I_{β} phases.

Table S1. Detailed list of compounds attained by pyrolysis analysis (% of total chromatographic area).

Peak nº	Compound	Origin	Cel_{Eglo}	Cel_{EgloT}	Cel_{Epro}	Cel_{EproT}
1	2-oxo-propanal	LM	3.0	2.2	2.2	1.9
2	2-butenone	LM	0.5	n.d	0.3	0.2
3	2,3-butanedione	LM	0.5	0.3	0.4	0.5
4	3-pentanone	LM	0.2	n.d	n.d	n.d
5	hydroxyacetaldehyde	LM	5.6	3.8	4.2	4.1
6	acetic acid + NI	LM	0.7	0.2	0.5	0.5
7	ethyl propenyl ether	LM	0.3	n.d	0.1	0.1
8	acetol	LM	2.3	0.5	0.6	0.7
9	CH_2=CH-CO-O-CH_3	LM	0.2	0.2	0.1	0.2
10	1,2-ethanediol	LM	0.5	0.1	n.d	n.d
11	3-hydroxypropanal	LM	0.8	0.2	0.2	0.2
12	1,5-(hydroxymethyl)-2(5*H*)-furanone	F	0.2	0.1	0.1	0.1
13	2(5*H*)-furanone	F	0.3	0.1	0.1	0.1
14	CH_2=CH-CO-CHO	LM	0.3	0.4	0.4	0.4
15	3-furaldehyde	F	0.3	0.4	0.4	0.4
16	CH_3-CO-CHOH-CHO	LM	n.d	0.3	0.2	0.3
17	CHO-CH_2-CH_2-CHO	LM	1.8	0.3	0.2	0.3
18	furfural	F	0.8	0.5	0.7	0.6
19	2-cyclopenten-1-one	O	0.8	0.5	0.7	0.6
20	5-methyl-2(3*H*)-furanone	F	n.d	0.1	0.1	0.1
21	furfuryl alcohol	F	0.3	0.1	0.1	0.1
22	2-methyl-2-cyclopenten-1-one	O	0.2	n.d	n.d	n.d
23	2-cyclopentene-1,4-dione	O	0.2	0.1	0.1	0.1
24	dihydro-4-hydroxy-2(3*H*)-furanone	F	0.3	0.3	0.4	0.3
25	2-hydroxy-2-cyclopenten-1-one	O	1.9	0.3	0.6	0.6
26	dihydro-methyl furanone isomer	F	0.5	0.3	0.3	0.3
27	5-methyl-2-furaldehyde	F	0.4	0.2	0.2	0.2
28	NI sugar	O	n.d	0.1	0.2	0.2
29	3-methyl-2-cyclopenten-1-one	O	0.1	n.d	n.d	n.d
30	dihydro-2(3*H*)-furanone.	F	0.1	n.d	0.1	0.1
31	2(5*H*)-furanone	F	0.6	0.2	0.2	0.2
32	4-hydroxy-5,6-dihydro-(2*H*)-pyran-2-one	P	0.3	0.4	0.5	0.5
33	2*H*-pyran-2-one	P	0.3	0.3	0.3	n.d
34	3-methyl-1,2-cyclopentanedione	O	1.1	0.2	0.2	n.d
35	methyl-dihydro-(2*H*)-pyran-2-one	P	n.d	0.4	0.7	0.3
36	2-hydroxy-1-methyl-1-cyclopentene-3-one	O	0.6	n.d	n.d	0.5
37	phenol	L	0.1	0.1	0.1	0.1
38	guaiacol	L	n.d	n.d	0.1	0.1
39	2,5-dimethylfuran-3,4(2*H*,5*H*)-dione	F	0.2	0.2	0.2	0.4
40	*cis*-3-cyclopentene-1,2-diol	O	0.2	0.1	0.1	0.1

41	o-cresol	L	n.d	n.d	n.d	0.1
42	3-ethyl-2-hydroxy-2-cyclopenten-1-one	O	0.1	n.d	n.d	n.d
43	NI	F	0.4	0.2	0.5	0.3
44	3-hydroxy-2-methyl-(4*H*)-pyran-4-one (maltol)	P	0.4	0.2	0.1	0.3
45	2,5-furandicarboxaldehyde	F	0.3	0.2	0.2	0.2
46	4-methyl-(5*H*)-furan-2-one	F	0.2	0.1	0.1	n.d
47	1,5-(hydroxymethyl)dihydro-2(3*H*)-furanone	F	0.3	n.d	n.d	0.2
48	levoglucosenone	P	n.d	0.5	0.7	0.5
49	creosol	L	n.d	n.d	0.1	0.6
50	NI sugar	O	n.d	n.d	0.1	n.d
51	DL-arabinose	O	n.d	n.d	0.2	n.d
52	3,5-dihydroxy-2-methyl-(4*H*)-pyran-4-one	P	0.2	0.4	0.4	0.7
53	NI sugar	O	1.3	1.6	2.0	1.7
54	similar to dihydro-6-methyl-(2*H*)-pyran-3(4*H*)-one	P	0.3	n.d	n.d	n.d
55	3,4-anhydro-D-galactosan	O	0.7	0.5	0.2	0.5
56	1,4:3,6-dianhydro-α-D-glucopyranose	P	0.6	1.2	1.1	1.0
57	4-vinylguaiacol	L	n.d	n.d	n.d	0.4
58	NI sugar	O	0.2	0.3	0.4	0.4
59	5,6-dihydro-4-methoxy-(2*H*)-pyran	P	n.d.	0.5	0.3	0.5
60	2,3-anhydro-d-mannosan	O	0.2	0.2	0.2	0.4
61	D-fucose	O	n.d	n.d	0.2	n.d
62	5-hydroxymethylfurfural	F	1.4	1.9	1.5	2.1
63	3,4-anhydro-D-galactosan	O	0.3	0.4	0.4	0.0
64	syringol	L	n.d	n.d	0.1	0.8
65	NI sugar	O	n.d	0.2	0.3	n.d
66	2-hydroxymethyl-5-hydroxy-2,3-dihydro-(4*H*)-pyran-4-one	P	0.8	5.7	3.7	0.3
67	1,5-anhydro-arabinofuranose	O	0.4	0.4	0.4	0.4
68	4-methylsyringol	L	n.d	n.d	0.2	0.8
69	vanillin	L	n.d	n.d	n.d	0.2
70	similar to 3,4-anhydro-D-galactosan	O	0.4	n.d	n.d.	n.d
71	4-vinylsyringol	L	n.d	n.d	n.d	0.2
72	NI sugar	O	0.1	0.3	0.4	0.4
73	NI sugar	O	0.1	0.3	0.3	0.3
74	1,6-anhydro-β-D-glucopyranose (LG)	P	50.6	53.5	52.9	43.6
75	1,6-anhydro-α-D-galactofuranose	O	1.3	2.0	2.0	1.6
	**% identified compounds**		**87.2**	**84.3**	**85.0**	**74.3**

LM – low molecular compounds; F – furan; P – pyran; O – others; L – lignin derivatives. NI – not identified sugar; n.d. – not detected; LG – levoglucosan.
